# Using topological data analysis and pseudo time series to infer temporal phenotypes from electronic health records

**DOI:** 10.1016/j.artmed.2020.101930

**Published:** 2020-08

**Authors:** Arianna Dagliati, Nophar Geifman, Niels Peek, John H. Holmes, Lucia Sacchi, Riccardo Bellazzi, Seyed Erfan Sajjadi, Allan Tucker

**Affiliations:** a Centre for Health Informatics, University of Manchester, Manchester, United Kingdom; b Manchester Molecular Pathology Innovation Centre, University of Manchester, United Kingdom; c NIHR Manchester Biomedical Research Centre, University of Manchester, United Kingdom; d Department of Biostatistics, Epidemiology, and Informatics, Penn Institute for Biomedical Informatics, University of Pennsylvania Perelman School of Medicine, USA; e Department of Electrical, Computer & Biomedical Engineering University of Pavia, Italy; f Department of Computer Science, Brunel University London, United Kingdom

**Keywords:** Type 2 diabetes, Unsupervised machine learning, Longitudinal studies, Electronic phenotyping

## Abstract

Temporal phenotyping enables clinicians to better understand observable characteristics of a disease as it progresses. Modelling disease progression that captures interactions between phenotypes is inherently challenging. Temporal models that capture change in disease over time can identify the key features that characterize disease subtypes that underpin these trajectories. These models will enable clinicians to identify early warning signs of progression in specific sub-types and therefore to make informed decisions tailored to individual patients. In this paper, we explore two approaches to building temporal phenotypes based on the topology of data: topological data analysis and pseudo time-series. Using type 2 diabetes data, we show that the topological data analysis approach is able to identify disease trajectories and that pseudo time-series can infer a state space model characterized by transitions between hidden states that represent distinct temporal phenotypes. Both approaches highlight lipid profiles as key factors in distinguishing the phenotypes.

## Highlights

• Topological Data and Pseudo Time Series to discover Type 2 Diabetes temporal phenotypes.
• Temporal phenotypes inferred from state-space model based on hidden-states transitions.
• Study of states continuous transitions visually delivered in an easily explainable way.
• Mined phenotypes characterized by significant differences in disease deterioration.

## 1 Introduction

Electronic temporal phenotyping is the identification of clinically meaningful event sequences from patient data that have been collected over time. The identification of temporal phenotypes that are specific to subgroups of patients can assist researchers in identifying useful cohorts and could also be used to generate hypotheses for precision medicine research. What is more, they help experts to better understand the disease in question and how it progresses over time, while ensuring that existing guidelines and care plans are appropriate. An interesting set of methods recently used for temporal phenotyping is represented by temporal graphs extracted from electronic health records [1,2]. While time-series data are ideal for such investigations [3], these are not always readily available.

Unlike most previous research that is based on extracting phenotypes from longitudinal electronic health records, we are interested in the construction of temporal phenotypes based on the overall structure of data (that is not necessarily longitudinal) and the identification of realistic trajectories through this structure in time.

**Topological Data Analysis** (TDA), enables structural phenotype discovery from large, complex data by creating networks of individuals and linking those who display demographic, clinical, and biomarker similarities. TDA provides an analytic method for complex clinical and -omics data to identify shape characteristics that are robust to changes by rescaling distances resulting in a qualitative description of the data. Leveraging methods adapted from topological mathematics, which studies the characteristics of shapes that are not rigid, TDA approaches consider fundamental properties like coordinate invariance, deformation invariance and compression [4,5].

TDA captures the structure of shape in data by connecting related data points and building topological models as networks. This allows for visualization of a “disease space”, the underlying shape of the data, and the identification of relevant groupings as connected components of the network. A relevant feature of TDA is that it builds a continuous shape on top of the data, allowing to study patients’ conditions as a continuum, where subjects can fluctuate over the disease space, moving through the nodes of the network graph. TDA therefore differs from clustering. As it can effectively represent continuous variation. While TDA exploits hierarchical clustering in building its network graph, it adds additional precision to the groups that are formed. TDA avoids the need of clustering methods to break things apart even if they belong together, and local behaviors can be lost or obscured. This can be particularly problematic in data sets that contain progressions and where data are naturally connected (i.e. repeated observations from EHRs), On the contrary, topological projections represent geometric aspects beyond the breakup into clusters; as detailed in the methods section, TDA performs clustering within overlapping sections of the data set, preserving connections in the mined networks [6,7].

TDA provides intuitive representations of results, which are calculated using linear algebra and geometric parameters. Its simplicity and ease of interpretation responds to a current compelling challenge of artificial intelligence: to translate research results into transparent and accessible tools based on data visualization and interactive data exploration [8]. Algorithms underpinning TDA are well defined [5,9,10].

Topological Data Analysis (TDA) allows one to model complex data by focusing on capturing data shapes. While topology is a mathematical formalism for measuring and representing shapes, TDA uses topology in order to visualize and explore high dimensional and complex real-world data sets and represent them as network graphs. The mathematical tools to identify shape characteristics of data sets with topology are called topological mappers [6,19] and they work by identifying the shape of a data set along specific filter functions, as follows:

(1) The points in the dataset are represented with a similarity metric that measures the distance between points in the space;
(2) The filter functions (lenses) project the points into a coordinate space and describe the distribution of data in that space;
(3) The projections are partitioned into overlapping bins. The bins are defined by resolution, which sets the number of bins that are created within the projections’ range of selected lens values, and by gain, which defines the amount of overlap between bins;
(4) A clustering step is carried out within each of these bins. This step defines the geometric scale of the shape and is defined by the number of clusters in each bin;
(5) Finally, the network graph is generated by plotting clusters as the graph nodes where shared samples (between bins) are connected by an edge.

Once the graph is generated it is possible to color nodes and edges with the average value of filter functions or to generate a specific function that represents variables of interest (e.g. number of observations in the bins, average age of the subject in bins etc). These features make TDA a suitable tool to depict temporal phenotypes and the progression of diseases. Topology alone does not satisfy the temporal aspects of a dynamical system. However, topology, especially persistent homology, has been considered to deal with time delay embedding models in applications such as risk analysis and prediction of critical transitions in financial markets [11]. TDA has been also proposed for time series featurization without time-dependent structural assumptions on the data generating process [12].

In this work, we propose the joint use of pseudo time-series with TDA in order to illustrate the temporal characteristics of disease progression, so that disease trajectories can be constructed from the data using the topological model as a guide. A **pseudo time-series** (PTS) [13,14] exploits the characteristics of disease progression so that realistic trajectories can be constructed from cross-sectional data. It uses known labels that determine the beginning and endpoints of a trajectory so that a time-series can be created to better understand the metabolism or cell cycles in genomic data [15,16], or the different variations of progression in diseases such as glaucoma or cancer [17]. PTS has also been used to integrate longitudinal studies with cross-sectional data [18]. In contrast to other unsupervised methods that provide intuitive and easy to interpret visual results, such as Self Organizing Maps, TDA outputs fundamental features (i.e. coordinate invariance, deformation invariance) and structure as network graph allow the straightforward application of pseudo-time inference approach.

We focus on microvascular complications of type 2 diabetes mellitus (T2DM) and explore both TDA and PTS for building different trajectories from health record data in order to better understand the temporal phenotypes that can identify different sub-phenotypes of T2DM.

## 2 Methods

**Fig. 1:**
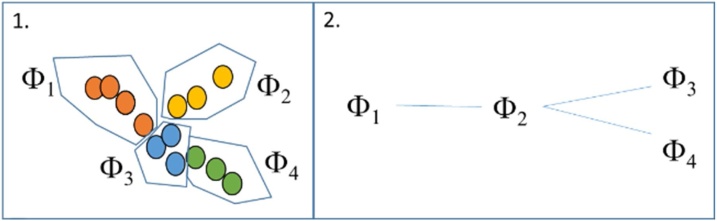
Methodological steps: 1. TDA finds sub-phenotypes, identified as φ. 2. Pseudo-time reconstruct transitions and trajectories to derive temporal phenotypes.

In the following, we describe our approach to discover T2DM temporal phenotypes (Fig. 1). First, we used TDA to identify subgroups of disease characteristics from cross-sectional record-level data, not ordered in time; we considered these as “sub-phenotypes”. TDA is used to identify an overall, complex structure with multiple trajectories by applying a minimum-spanning-tree filter, which identifies a number of feasible trajectories representing different temporal phenotypes. Second, we explored pseudo-time approaches, which involve using a combination of distance metrics and graph theory to reconstruct transitions among the phenotypes and infer realistic trajectories through the data space from early disease stages through to advanced ones.

### 2.1 Topological data analysis

We used the Topological Data Mapper implementation described in [5] to perform our analysis, and perform the analysis using the function mapper2D from Topological Data Analysis using Mapper R package [https://github.com/paultpearson/TDAmapper].

Parameterization of TDA. We used cosine distance in conjunction with single-value decomposition (SVD) and L1-infinity centrality (which assigns to each point the distance to the point most distant from it) as filter functions to build the topology. This is based on the same pipeline adopted in [20] and has been found to provide a more detailed and succinct description of the data than typical scatterplots. We explored the effect of varying resolution parameters (i.e., number of bins and their overlapping) and the geometric scale (i.e., the number of clusters within bins) and using a grid search. It is important to tune parameters and scale in order to insure a shape granularity fine enough to detect temporal behaviors (i.e. repeated observations in time of individual patients aren’t restrained within the same node). A too-coarse granularity would result in state changes within nodes, which might impede trajectory discovery.

Topology and clustering. The output of the TDA algorithm is a graph object that can be analysed with a network analysis package [http://igraph.org/]. In order to identify distinct topology sections that allow us to retrieve sub-groups of observations, we applied the cluster optimal function [21], which calculates the optimal community structure of a graph, by maximizing the modularity measure over all the possible partitions.

Minimum Spanning Tree on Topology. In order to identify specific trajectories from the overall topology, we applied a minimum spanning tree filter to detect the shortest paths within the topology. The weights were based on the average time of the observations represented in the topology’s edges. While temporal features were not used to retrieve the original topology, the minimum spanning tree was guided by time to illustrate disease temporal pathways.

Subject assignment to TDA trajectories. Similarity measures were used to compare and assign individual trajectories to the ones mined by TDA. TDA graph nodes can be seen as event data representing a model of progression across states, where each node is identified by a fixed index. Thus we compute Jaccard similarity to assign individual subjects to mined trajectories, as previously exploited in the context of careflow mining [22]. Jaccard similarity coefficients are computed between each sequence of events that build the individual trajectory and all of the detected trajectories (i.e. the temporal phenotypes).

For each i-subject trajectory Ti, Jaccard_id_ is computed to compare it to the all mined d-trajectory Td:

$$\text{J}\text{a}\text{c}\text{c}\text{a}\text{r}\text{d}\text{i}\text{d}\;(\text{T}\text{i},\text{T}\text{d})\;=\;\frac{\left|\text{T}\text{i}\;\cap \;\text{T}\text{d}\right|}{\left|\text{T}\text{i}\;\cup \;\text{T}\text{d}\right|}$$

Ti (i.e. each individual) is then compared and assigned to the trajectory Td with the highest Jaccard_id_.

For example, assuming that three trajectories are identified (Td_1,_ Td_2,_ Td_3_), reported below as sequence of node indices, individual trajectories (for example Ti for i-th subject) are compared to each Td via Jaccard similarity. In this example the i-th subject is assigned to Td3.

It is worth noting that, while in the original trajectory Ti* a subject could stay in a node for one or more consecutive observations from the original data set (e.g., i-subject stays in node 41 for five consecutive follow-ups), the mined trajectories only report the sequence of nodes. Therefore, the information regarding the follow-ups is not used in Ti to compute the Jaccard similarity.

Original Individual trajectory

Ti* = < 41^5^, 42^6^, 43^2^, 39^2^, 40^3^, 33^2^, 34^2^, 26^3^, 27^1^, 19^1^>

Individual trajectory

Ti = < 41, 42, 43, 39, 40, 33, 34, 26, 27, 19>

TDA mined trajectories and Jaccard similarity values

**Table:** 

	Jaccard similarity
Td_1_ = <41, 37, 30, 22, 15, 8, 1>	6.25 %
Td_2_ = <41, 42, 43, 39, 32, 24, 17, 10, 3>	26.67 %
Td_3_ = <41, 42, 43, 39, 40, 33, 34, 26, 19, 12, 5>	75 %

### 2.2 Pseudo-time series

Pseudo-time-series (PTS) methods can be used to infer state-space models that are characterized by transitions between explicit hidden states, representing distinct temporal phenotypes.

**Fig. 2:**
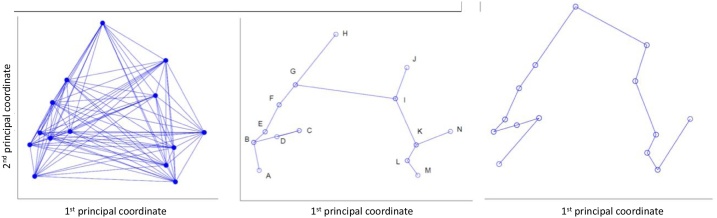
Generation of pseudo-time series from left to right: (a) the weighted graph of a sample of data (b) the minimum spanning tree of the weighted graph and (c) the Pseudo Time-Series.

The idea behind PTS is to exploit resampling, distance metrics, and assigned class labels to build realistic trajectories from one label state to another. Here we used microvascular complications as the class label. This is one of the main indicators of the progression of the disease for T2DM patients [23]. Firstly, a chosen distance metric is selected for calculating distance between all data points. Here we chose the cosine distance for direct comparison to the TDA. Resampling is used to generate multiple distance matrices from this complete matrix for a sub-sample of the data points. These data points are then used to build a weighted graph (see Fig. 2a) and the associated minimum spanning tree (Fig. 2b). Two randomly predefined points were identified as the start-point and endpoint of a trajectory within the tree. We accepted any sampled patient that has no microvascular complications as a potential start and any patient with microvascular complications as a potential endpoint. The shortest path was identified between the start-point and the endpoint within the minimum spanning tree, resulting in a single pseudo time-series (Fig. 2c). The entire resampling procedure was repeated 1000 times to generate multiple pseudo time-series.

These time-series can then be used in conjunction with the EM algorithm to infer state space models that capture the dynamics of the trajectories. This was implemented in MATLAB using the Bayes Net Toolbox [24]. The EM algorithm was used to infer parameters and the junction tree algorithm to perform inference within an autoregressive Hidden Markov model.

### 2.3 Evaluation on simulated data

Evaluation of the joint utility of the two methods has been carried out. We used data simulated via PTS methods to derive topologies, in order to provide a more robust evidence for approaches that combine the two methods.

We generated a set of simulated observations using a hidden Markov model with five underlying hidden states that control the topology. We then performed TDA on the simulated data to explore the capability of the topology in capturing the original hidden states and transitions between them, thus confirming TDA capability of reconstructing temporal pathways.

### 2.4 Comparison with baseline methods

In [19,25], TDA results were compared with alternative unsupervised machine learning methods, including clustering and Principal Components Analysis (PCA). Indeed, TDA can be described as a method combining clustering approaches (hierarchical clustering is used to derive nodes) and PCA (lens function and their projection in a geometrical space can be considered as the components to study the correlation structure of the data).

While previous comparisons of TDA techniques with baseline methods were performed on cross-sectional data, this work embeds the temporal dimension with pseudo time techniques, which are not exploited in the comparison, thus omitting the information about temporal pathways. Here we compared TDA results with Self-Organizing Maps (SOMs) [26] ones. SOMs are unsupervised approach that allow a discretized representation of the input space preserving its topological properties thus they seemed the most appropriate approach for the temporal projection of data and a qualitative comparison with temporal enriched TDA.

### 2.5 MOSAIC data

Data for this study was previously collected for clinical and management purposes during the MOSAIC project funded by the European Commission under the 7th Framework Program, (Theme Virtual Physiological Human, 2013–2016) [22,27,28]. Health records were accumulated from 924 pre-diagnosed T2DM patients, which resulted in 13,623 instances in our data set. Risk factors found to influence T2DM [28] include: body mass index (BMI), systolic blood pressure (SBP), diastolic blood pressure (DBP), high-density lipoprotein (HDL), triglycerides, glycated hemoglobin (HbA1c), total cholesterol and smoking habits. Accordingly with previous studies on the MOSAIC project [28], the experimental results were mined for microvascular comorbidities (diabetic nephropathy, neuropathy, and retinopathy).The following variables were used to build the topology and pseudo time-series: age, smoking habit, HbA1c, BMI, SBP, total cholesterol, and triglycerides. Continuous variables were normalized on a -1 to +1 scale. While we did not exploit the temporal nature of this data for phenotype identification, we used the fact that many of these patients had varying follow-up measurements to evaluate our trajectories. In particular, we used time-since-first-visit to assess whether the trajectories correctly model patient progression.

Microvascular comorbidities onset was contrasted in subjects belonging to the discovered trajectories using Kaplan Meier visualization. Given the results obtained by the Kaplan-Meier analyses, we investigated whether the mined patients’ groups were significant predictors of the onset of microvascular complications if we consider also the available clinical variables in a statistical model. To this end, we have carried out a multivariate survival analysis by using Cox-Regression to predict onset probabilities.

## 3 Results

### 3.1 Topological data analysis

**Fig. 3:**
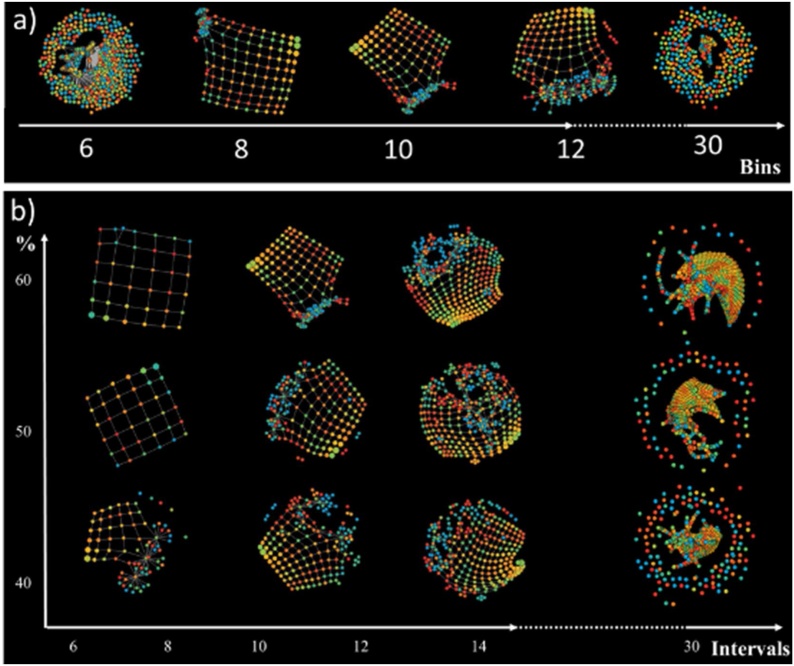
Topologies a) varying Geometric Scale and b) varying Resolution Scale and Percentage Cluster Overlap (gain).

**Fig. 4:**
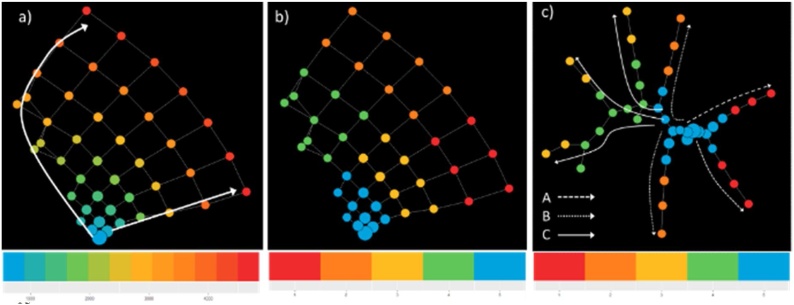
The network retrieved via TDA and displayed with igraph. In a) nodes are coloured by time from the first visit, in b) with the cluster membership. In c) The Minimum Spanning Tree identifies trajectories of patients. The node colouring is based upon the clustering membership. (For interpretation of the references to colour in this figure legend, the reader is referred to the web version of this article).

**Fig. 5:**
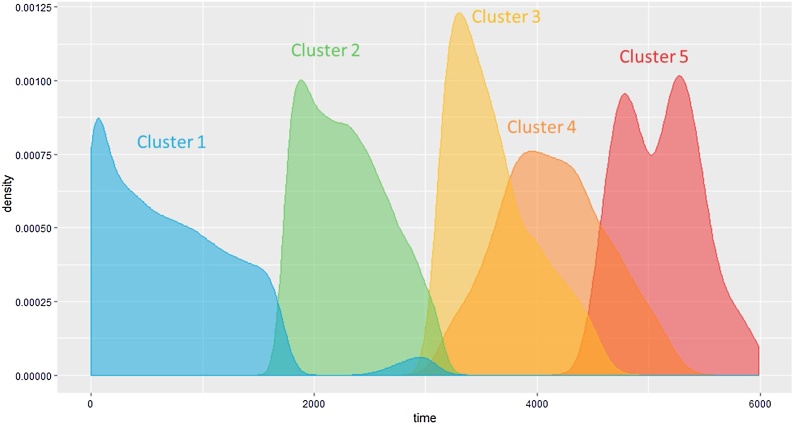
Follow-up time distributions in the optimal community structure clusters.

The graphs in Fig. 3, Fig. 4 illustrate the result produced by the TDA algorithm. Each node represents a cluster of data points as observations in time (i.e., an encounter in the MOSAIC data set). The nodes are coloured with the time (days) from the first visit of each encounter. Fig. 5a reports the distribution of the value on a continuous colour scale from blue (time = 0 days from the first visit) to red (time = 4000 days from the first visit).

First, we explored the effect of varying the number of clusters within each bin, which defines the geometric scale of the topology (Fig. 3a). In general, a lower value results in very small clusters (sometimes individual data points), and for higher values the network starts to become extremely sparse or loosely connected. In both cases, edges, which are based on shared samples, are impossible to extract and resulting shapes don’t show any relevant topological features. Fig. 3a demonstrates a relatively stable topology for between eight and 12 clusters per bin. For the remainder of the analysis we chose a value of 10. Secondly, we explored the resolution scale while also varying the degree of cluster overlap (gain) when determining the topology (Fig. 3b). In general, higher gain results in more edges. Increasing the resolution of a graph increases the number of bins. In Fig. 3b the horizontal axis represents the number of overlapping intervals and the vertical axis represents the percentage overlap. Note that while the percentage doesn't affect the shape considerably, the interval sizes between 6 and 14 enable a stable shape. For higher values, the network becomes too unstable and it is more difficult to recognize any characteristic shape or trajectories within the network.

Fig. 4 illustrates a stable topology generated with seven bins, 60 % overlap, and a geometric scale of 8; this is the one used in the following analysis steps. Fig. 4a reports the topology enriched by time from the first visit, whose distribution is given in the bottom panel. It is possible to identify a clear temporal direction from the blue bottom node towards the red nodes, thus indicating that the temporal progression itself can be reconstructed by TDA. Furthermore, Fig. 4b reports the topology enriched by the five clusters obtained applying the optimal community structure cluster on TDA results. Fig. 5 illustrates the follow-up time distributions in each cluster, indicating how TDA is able to reconstruct clusters’ temporal progression (see the increase in time density distributions form Cluster 1 to Cluster 5), even if the time-dependent structure of the generating process is not explicitly represented in the input.

The minimum spanning tree identified seven distinct trajectories (Fig. 4c); all of which start from the central blue cluster which accounts for the first observations in time. We manually grouped the mined trajectories on the basis of their final state as follows: A) the two trajectories that lead to the red clusters, B) the two trajectories that lead to the orange clusters and C) the three trajectories that lead to the yellow clusters past the green clusters. These three groups represent disease progression phenotypes, which we refer to as temporal phenotypes.

### 3.2 Pseudo-time trajectories

As previously shown, Fig. 2a reports the weighted graph constructed on the basis of a cosine distance. This graph was used to construct the minimum spanning tree (Fig. 2b). Randomly predefined points were identified as the start-point and endpoint of a trajectory. One data point classed as having no microvascular complications was randomly selected as a starting point, and one data point classed as having at least one microvascular complication is randomly selected as an end point.

**Fig. 6:**
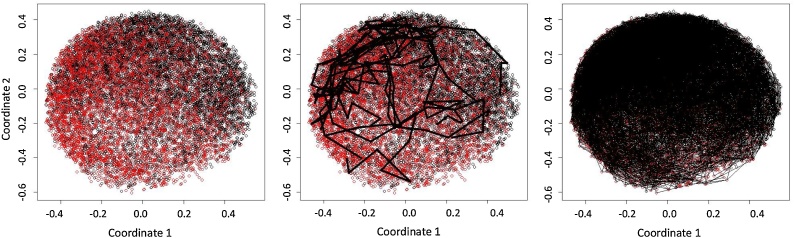
Left: Multidimensional Scale plot of Cosine Distance where red represents patients with at least one microvascular complication, and black represents none. Middle: Cosine Plot with 10 sample Pseudo-time Series trajectories plotted, right: Full 1000 Pseudo-time Series Generated. (For interpretation of the references to colour in this figure legend, the reader is referred to the web version of this article).

The shortest path was identified between the start-point and the endpoint within the minimum spanning tree, resulting in a single pseudo time-series (Fig. 2c). The entire resampling procedure was repeated 1000 times to generate multiple pseudo time-series. Fig. 6 illustrates the cosine distance plot enriched with the information about having developed or not a micro vascular complication during the observation period. The pseudo time series (10 samples and all of them) have been plotted upon the graph showing the correlation between trajectories of disease and complications. Having constructed 1000 pseudo-time series, we used an Autoregressive Hidden Markov Model (ARHMM) with 5 discrete hidden states to build a model to capture the dynamics of the different trajectories through the data. The 5 underlying classes were selected based upon experimentation of a number of clinical datasets using PTS methods in [17].

### 3.3 Clinical assessment

Using data from T2DM patients, we created a topological data network, selecting the network with the most stable topology, and enriched the topology with time-from-the-first-visit information. This process revealed potential trajectories for disease progression (Fig. 3a) and sub-groups of observations from the topology clustering (Fig. 3b). Having identified the most suitable topology, the graph was used to build a minimum spanning tree in order to identify pseudo-time-based trajectories (Fig. 3c). Using this approach, seven potential trajectories were identified. These trajectories have been grouped in three temporal phenotypes: A, B and C (Fig. 3c), which show the progression of each trajectory (each one representing a T2DM temporal phenotype) towards the disease’s deterioration and distinct outcomes.

**Fig. 7:**
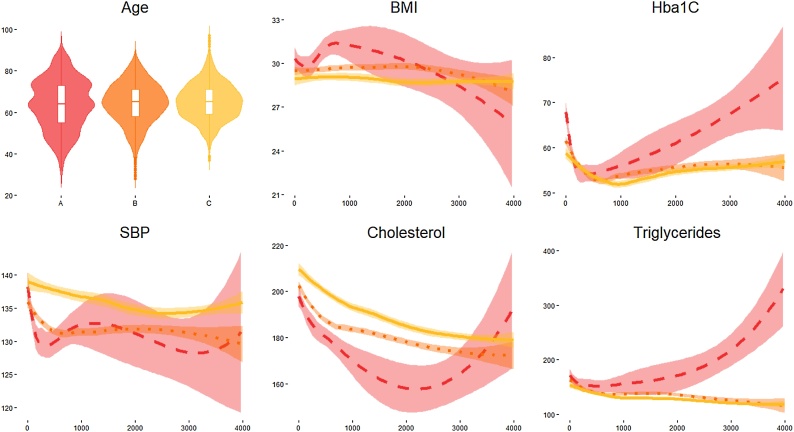
Clinical characteristics over time of subjects in the A (red-dashed), B (orange-dotted) and C trajectories (yellow-continuous) – in all he panels, x-axis indicate time, in days, from the first visit. Values of the y-axis indicate Age in years, BMI in kg/m^2^, Hba1C in mmol/mol, SBP in mm Hg, Cholesterol and Triglycerides in mg/dL. (For interpretation of the references to colour in this figure legend, the reader is referred to the web version of this article).

We characterize these phenotypes using relevant clinical features values at baseline (Table 1), and as they develop in time (Fig. 7). Patients belonging to the C phenotype demonstrated higher cholesterol levels and systolic blood pressure at baseline and over time. Further, the A phenotype shows a higher and increasing level of HbA1c, a decreasing and then increasing trend of cholesterol, and an increasing trend of triglycerides.

**Table 1:** Baseline characteristics – continuous variables are compared with ANOVA, Time from Diagnosis and Triglycerides by Kruskal Wallis test and gender by chi-square.

	Temporal Phenotype			p-value
	A	B	C	
Total Number of Subjects	191	574	159	
Gender Male - N(%)	107 (56 %)	340 (59.1 %)	94 (59.1 %)	0.727
Age - Mean (SD)	63.99(12.07)	64.43(9.99)	66.18(9)	0.007
Time from Diagnosis – Median (IQR)	4.81(13.1)	7.1(10.3)	10.1(10.2)	0.497
Hba1c - Mean (SD)	58.63(16.8)	55.28(15.61)	54.99(12.79)	0.547
BMI - Mean (SD)	30.27(6.06)	29.6(4.94)	28.8(4.54)	0.288
Cholesterol - Mean (SD)	184.94(36.31)	185.53(31.56)	186.96(29.24)	<0.001
Triglycerides - – Median (IQR)	139(77.8)	127(63.3)	119(51.5)	0.20
SBP - Mean (SD)	131.98 (17)	132.16 (14.12)	135.4 (14.04)	0.008

**Fig. 8:**
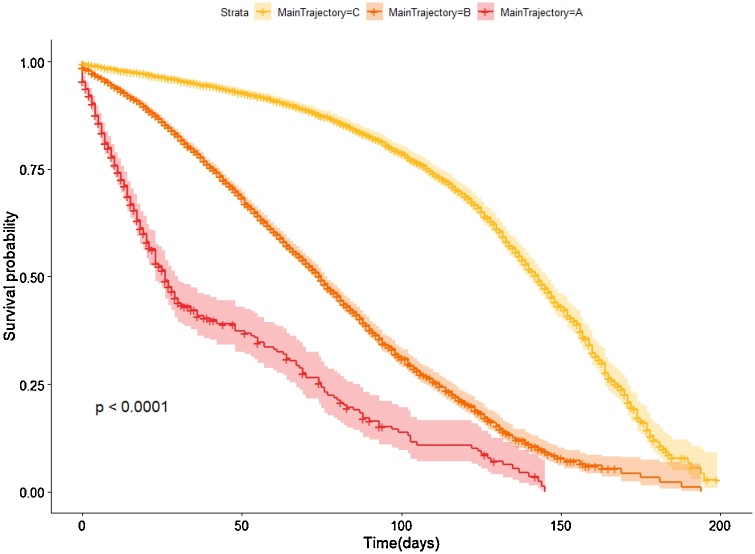
Kaplan Meier curves having the onset of micro vascular complication as endpoints.

Temporal phenotypes were compared in terms of microvascular complications’ onset. We considered the registered onset date of microvascular complications as main endpoint, and Kaplan-Meier analysis finds a statistically significant difference (p < 0.0001) among temporal phenotypes. Looking at complications disease-free survival (Fig. 8), the group with the worst prognosis is represented by phenotype A. Therefore, minimum spanning tree paths can identify groups of patients more (A phenotype) or less (C phenotype) exposed to the development of T2DM-related complications over time.

**Fig. 9:**
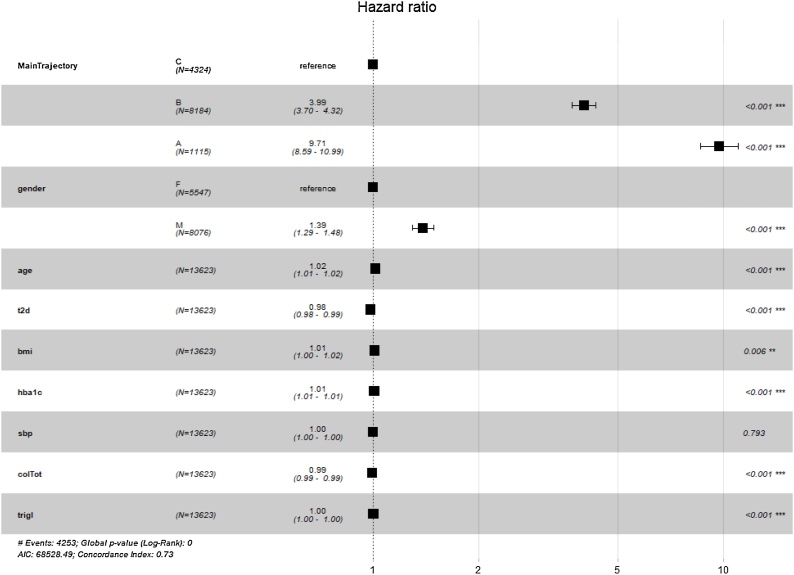
Cox Regression results reported as Hazard Ratio + 95 % Confidence Interval for Hazard Ratio and significance codes for the onset of microvascular complications.

We further investigated whether the phenotypes were significant predictors of the onset of microvascular complications by Cox-Regression. Results in terms of Hazard Ratios (HR) are shown in Fig. 9. It is possible to note that the mined temporal phenotypes are significant predictors of complications, even when adjusting for clinical data, where subject assigned to the phenotypes identified by the A trajectories have a significant higher risk (p < 0.001, HR = 9.71, Confidence Interval = 8.59, 10.99) when compared to C trajectories.

We now turn to the pseudo-time approach where we have inferred a five-state Auto Regressive Hidden Markov Model from the 1000 pseudo time-series generated from the original data. Table 2illustrates the expected values for the key features of the data for each of the five hidden states.

**Table 2:** Expected values for the five hidden states, where t2d represents time-since-first-visit, TotChol represents total cholesterol and Trigl represents triglycerides. Highest values are indicated in boldface.

State	1	2	3	4	5
% Female	0	0	0	50	**59**
% Male	100	100	100	50	41
Age	59.16	**69.41**	63.7	**67.78**	56
t2d	3.77	9.76	**13.4**	**11.86**	5.42
HbA1c	47.66	50.3	**62.6**	53.54	**60.7**
BMI	28.1	27.58	30.07	**30.31**	**31.02**
SBP	129.59	129.5	**136.08**	**134.8**	132.73
TotChol	187.51	167.28	183.7	**188.86**	**207.62**
Trigl	126.98	108.13	**136.71**	124.38	**232.46**

Looking at the expected statistics in Table 2, State 1 represents younger patients who have the shortest period of time since their first visit, State 2 represents the oldest patients, State 3 represents people with the highest Hba1c and SBP values and are the patients who have been visiting for the longest of time since their first visit, State 4 represents older patients who have been visiting for a relatively long period, while State 5 represents the youngest patients with the highest BMI.

**Fig. 10:**
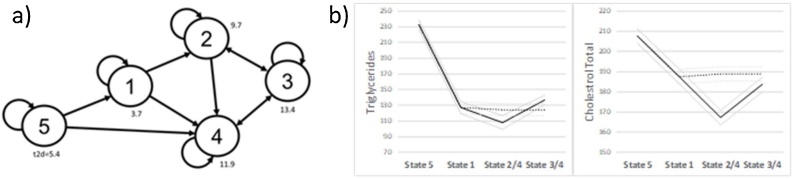
a) transition Diagram with expected time since first visit. b) Mean statistics for two trajectories 5-1-4 (dashed) and 5-1-2-3 (solid) for Triglycerides (left) and Cholesterol (right).

Table 3 illustrates the transition probabilities between these states. The transition probabilities in Table 3 indicate that all states are far more likely to remain the same than to change. The highest transition probabilities from one state to another are reported in bold. This is presented as a diagram in Fig. 10a, which captures a natural flow from State 5 to two potential End-States 3 and 4. This flow is supported by a general increase in the expected time-since-first-visit (t2d) shown in the diagram, as well as increasing age. End-State 3 represents patients with very high hb1ac and relatively lower cholesterol whereas End-State 4 captures older patients with relatively higher cholesterol but lower hb1ac and very low triglyceride levels. Fig. 10b shows two potential trajectories in the form of state transitions based on the HMM model: State transitions 5-1-4 and 5-1-2-3, for patients' triglycerides (left) and cholesterol (right). It is interesting to note that the lipid profiles were discovered as a defining characteristic of the two trajectories, similar to the TDA results in Fig. 7.

**Table 3:** State transition matrix.

	State 1	State 2	State 3	State 4	State 5
State 1	*0.733*	**0.145**	0.023	0.084	0.015
State 2	0.036	*0.866*	0.049	0.049	0
State 3	0.055	**0.127**	*0.678*	**0.132**	0.007
State 4	**0.159**	**0.113**	**0.157**	*0.542*	0.029
State 5	**0.134**	0	**0.107**	**0.140**	*0.618*

### 3.4 Simulated data

In order to assess the joint use of pseudo time approaches with TDA methods, and the capability of the latter to catch temporal progressions, we performed a set of analyses on longitudinal simulated data (7500 observations) generated from an autoregressive hidden Markov model with five underlying hidden states and two observed variables, *X* and *Y*.

**Fig. 11:**
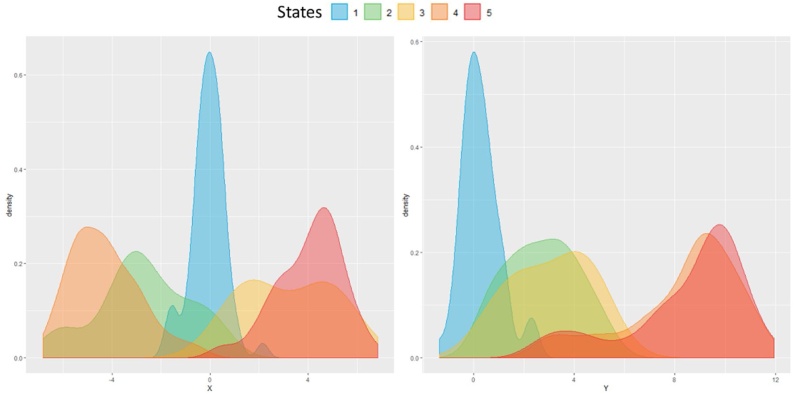
Simulated variables X and Y distributions in Hidden States. (For interpretation of the references to colour in this figure legend, the reader is referred to the web version of this article).

**Fig. 12:**
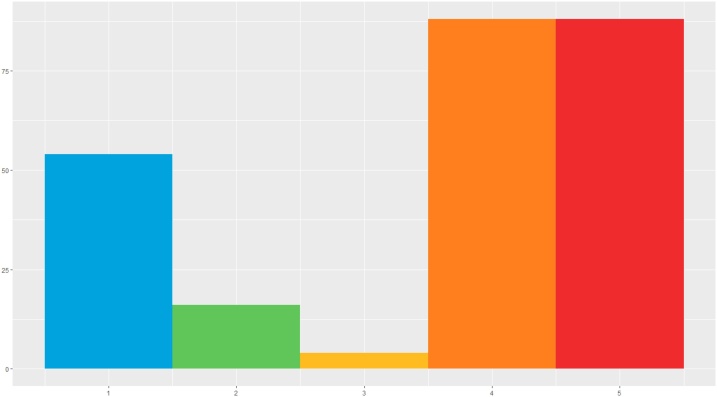
State distribution in the simulated data.

The transition probabilities were hand-coded into the model as shown in Table 4, whilst the initial and emission probabilities were hand-coded to ensure a realistic progression from the initial starting state (State 1) to two potential endpoints (States 4 and 5). The distributions of the generated data for X and Y for each of the five hidden states are reported in Fig. 11, the hidden state distribution in Fig. 12.

**Table 4:** State transition matrix in the simulated data.

	State 1	State 2	State 3	State 4	State 5
State 1	0.8	0.05	0.05	0.05	0.05
State 2	0.1	0.8	0	0.1	0
State 3	0.1	0	0.8	0	0.1
State 4	0	0	0	1	0
State 5	0	0	0	0	1

Given the structure of the simulated data, we run TDA using Euclidean distance and *X* and *Y* as functions. Node enrichment was then performed indicating the most frequent hidden state in the node. TDA parameter selection was performed with grid search as in the original application, but with slightly different intervals due to differing numbers of observations: between six and 10 clusters per bin to define the Geometric scale, between five and 10 intervals and between 40 % and 60 % of overlap for the resolution scale. We applied a MST filter with weights based on the index of the nodes, indeed indexes reflect the “topological proximity” of the nodes and the monotonicity of the functions in the simulated data.

While we had to compare TDA results with the model that generated the simulation, we fixed some further criteria for parameters selection, requiring:

• Fully connected networks (i.e. having number of components equal to one);
• Hidden States’ distributions in nodes similar to the original distribution (compared via Pearson correlation);
• Average path lengths close to 5, as the number of hidden states.

**Fig. 13:**
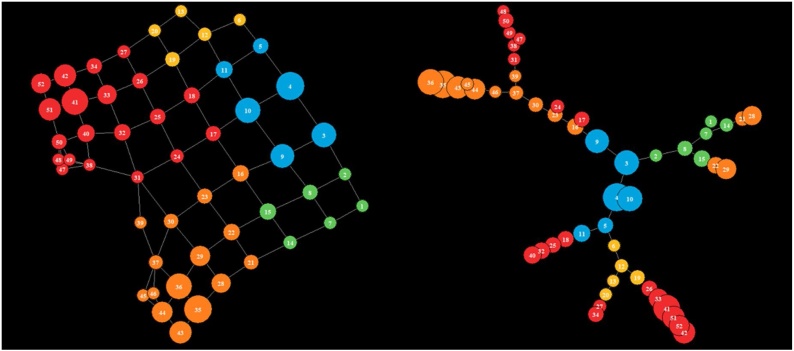
Topology and MST trajectories on Simulated data. Both are enriched with the majority Hidden State in each node. (For interpretation of the references to colour in this figure legend, the reader is referred to the web version of this article).

Table 5 reports the top 10 parameters combinations with highest values of Pearson correlation in fully connected networks. Fig. 13 illustrates the topology and MST obtained with 7 Intervals, 6 Bins and an overlap percentage of 50 %.

**Table 5:** TDA parameters combinations together with Distribution criteria and Average Path Length.

Number of Intervals	Overlap Percentage	Number of Bins	Hidden States Distribution - Pearson Correlation with original distribution	Average Path Length
7	50	6	0.9449133	5.167059
7	45	6	0.9408602	5.596531
8	55	6	0.934875	5.828645
7	60	6	0.9291068	5.517241
7	40	6	0.9231947	5.682504
7	55	6	0.9135777	5.167677
7	60	8	0.9076339	5.308756
8	60	6	0.8985553	5.930751
7	55	8	0.877915	5.523086
8	60	8	0.8769934	5.983423

While the capability of the topology in capturing hidden states was somewhat supported by choosing a set of parameters able to reproduce the Hidden State distribution in the TDA edges, the trajectories, which were derived from the MST exclusively on the basis of topological features, closely reproduce the transitions among Hidden States.

**Fig. 14:**
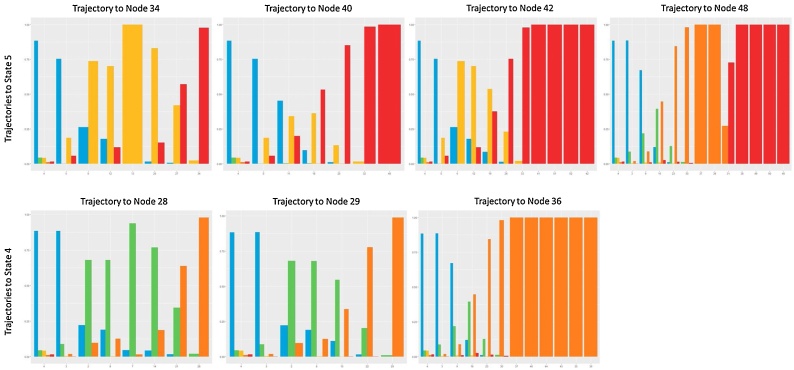
Hidden states distribution in the MST trajectories, progressing through the topology. (For interpretation of the references to colour in this figure legend, the reader is referred to the web version of this article).

On the left side of Fig. 13 is possible to observe that out of the 52 edges modelled by TDA the majority of them belong to Hidden State 4 (n = 15, % = 28.8) or State 5 (n = 20, % = 38.46), while they are quite evenly distributed in the remaining ones. More interestingly, on the right side of Fig. 13 and in Fig. 14, is possible to note how the MST is able to retrieve 7 trajectories: all of them progress from the initial State 1(in blue) to two potential end-States 5 (in red), and 4 (in orange).

Comparing these results with the transition matrix in Table 4, is possible to observe that the topology not only identifies the same end-States, but it also captures similar transitions among Hidden States:

• Trajectories from State 1 (blue) to State 3 (yellow) to 5 (red) in Fig. 13, which are also indicated by the distributions of states in nodes from 4 to 42, 34, and 40 in the histograms in the top part of Fig. 13.
• Trajectories from State 1 (blue) to State 2 (green) to 4 (orange) in Fig. 13, and in histograms in the bottom part in Fig. 13, with nodes from 4 to 28, 29, and 36.

### 3.5 Comparison with SOMs

**Fig. 15:**
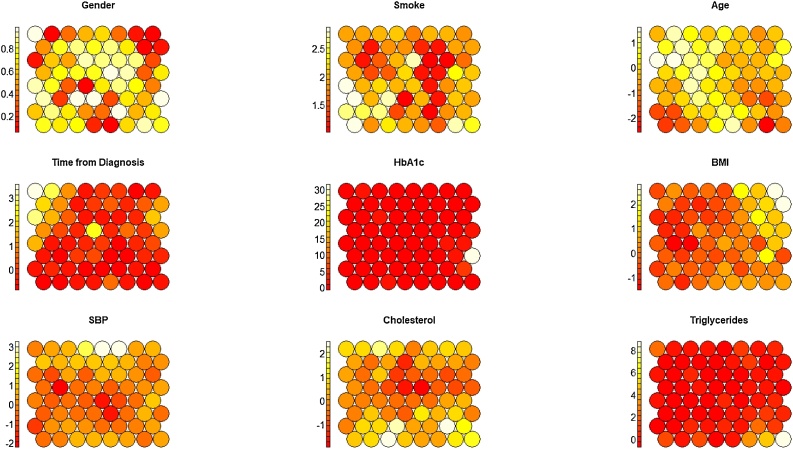
SOMs results: Kohonen Heatmap colored with clinical variables mean values. (For interpretation of the references to colour in this figure legend, the reader is referred to the web version of this article).

We compare TDA results with cluster analysis based on SOM. In order to reproduce a similar granularity and replicate the same geometric scale of the TDA, we choose a SOM grid of 8 × 8 nodes. Results of the Kohonen Heatmap for each of the variable used to build the map are shown in Fig. 15.

**Fig. 16:**
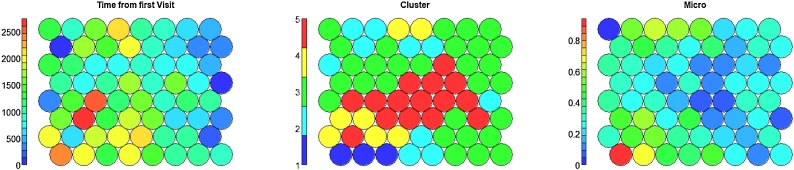
SOM results enriched with time, derived cluster and Micro vascular complications as outcome.

In order to compare the outputs of SOM with the Topological Mapper, we also provide Kohonen Heatmap coloured with (i) the temporal dimension used to construct TDA trajectories, (ii) the results of hierarchical clustering on the SOM codebook vectors (with a fixed number of clusters of five as in TDA) and (iii) the presence of microvascular complications in Fig. 16.

**Fig. 17:**
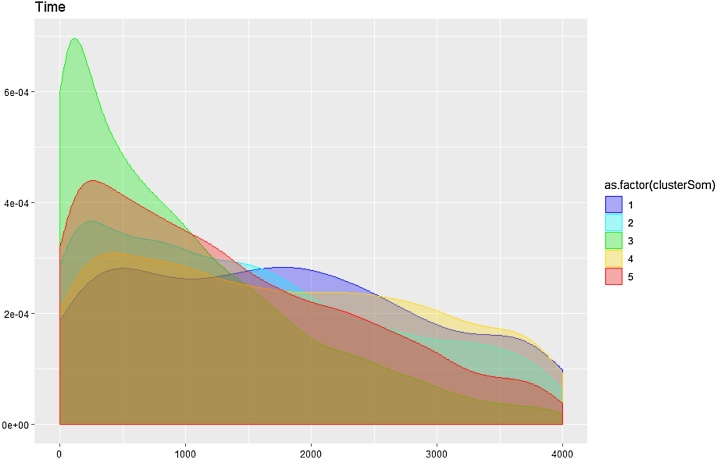
Follow-up time distributions in the SOM clusters.

Unlike the TDA, where it was possible to enhance the topologies with pseudo-time inference and represent the temporal dimension as a set of initial points concentrated in the topology, that then spread across the final ones (Fig. 5), SOM clusters identify compact sets of longitudinal observations (Fig. 17), thus failing to reconstruct a well-defined temporal progression from the clusters.

**Fig. 18:**
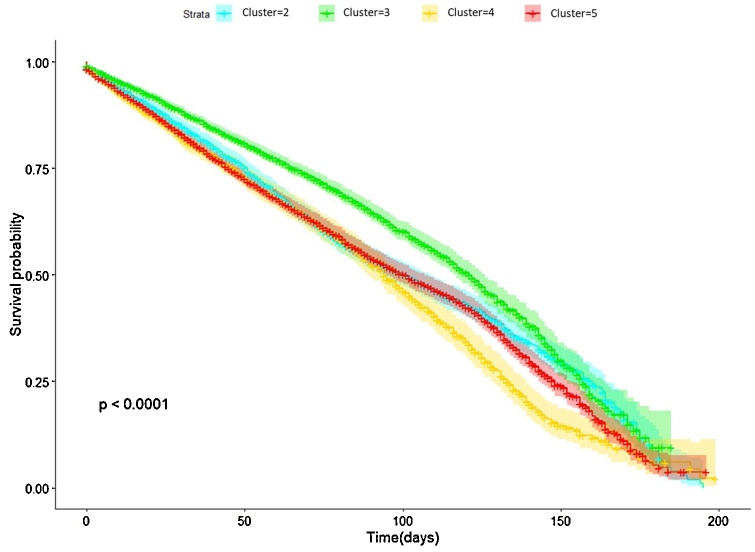
Kaplan Meier curves having the onset of micro vascular complication as endpoints in SOM clusters.

**Fig. 19:**
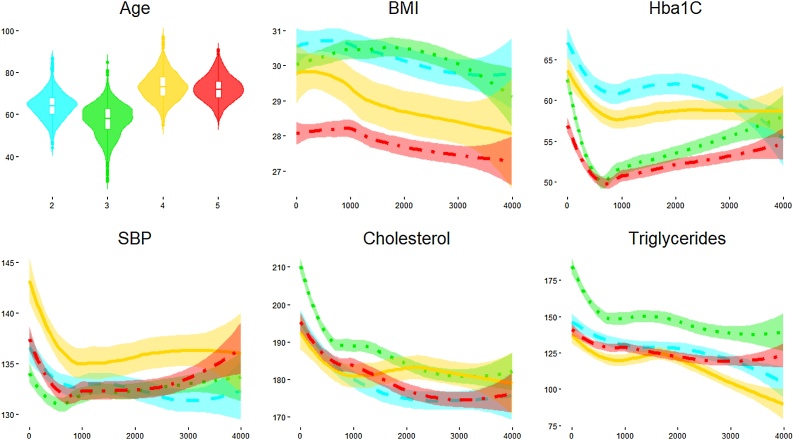
Clinical characteristics over time of subjects in SOM clusters– in all he panels, x-axis indicate time, in days, from the first visit. Values of the y-axis indicate Age in years, BMI in kg/m^2^, Hba1C in mmol/mol, SBP in mm Hg, Cholesterol and Triglycerides in mg/dL. (For interpretation of the references to colour in this figure legend, the reader is referred to the web version of this article).

Although SOM clusters are not able to identify temporal sequences of states (they do not represent temporal phenotypes), we assigned to each subject his/her majority cluster over the whole observation period (i.e. the cluster accounting for the highest number of observations) to validate of SOM results. We compared clusters by mean of microvascular complications (Fig. 18) and clinical characteristics (Fig. 19).

While results suggest that SOM are not able to clearly capture disease progressions as temporal phenotypes, the mined clusters have significant difference in terms of complications onset. In particular, the group with the best prognosis in term of complications disease-free survival is represented by cluster 3. Notably, subjects assigned to this cluster are the youngest, they have high but stable measures of BMI, decreasing and then increasing values of HbA1c and higher values of Triglycerides as compared to the other clusters.

## 4 Discussion

In this paper we present a comparison of two approaches to automatically building temporal phenotypes from electronic health records. TDA has been used to capture the overall shape of the data and a minimum spanning tree filter was applied to identify different trajectories. This approach highlighted subcategories of T2DM including one sub-cohort that displays different levels of cholesterol and initial Hba1c from the rest of the population. We also explored the use of PTS methods where different trajectories have been bootstrapped from the data and a state-space model was learned with five hidden states. This approach has identified only two trajectories; however, these are clinically relevant and support the findings made using TDA.

Neither TDA nor PTS relied on temporal features of the data in the health records to build these models. As a result, both approaches could be used to construct temporal phenotypes from cross-sectional data if appropriate disease staging information is included. Here we used microvascular comorbidity data, but any data that helps to stage a disease could be used. Both TDA and PTS show that in the studied population subjects tend to be relatively stable (they stay in Cluster 1, Status 1) – although subjects follow specific trajectories in deterioration of the disease, in this case measured as onset of micro vascular complications.

Baseline clinical values in the temporal phenotypes are comparable; however, their evolution in time differ. In particular, one of the mined trajectories (trajectory A) indicates a significant and rapid disease progression and higher risk in developing complications, which can also be seen in increasing values of HbA1c and triglycerides.

The robustness of the analysis pipeline has been validated following an inverse approach, where TDA was applied on data simulated via PTS. Results shown that TDA was able to track and reconstruct transitions among hidden states, thus indicating that the topology is able to embed and efficiently reconstruct pseudo temporal dimensions. On the contrary, when we analysed the same data via SOM, it wasn’t possible to retrieve well-defined temporal pathways, neither to map the relevant temporal phenotypes.

In this work, topologies are selected on the basis of grid search and qualitative evaluations. Further efforts are needed to classify the mined topologies and to compare different TDA parameter sets, in order to assess the stability of the results in a more rigorous and quantitative way. This can be achieved either studying the properties of topological stability, or by exploiting graph properties and invariants.

While the trajectories are derived from the exploitation of MSTs, a manual step is performed in order to aggregate the mined trajectories in temporal phenotypes (i.e. form 7 trajectories to 3 phenotypes based on the attractor final state). Further work is needed to embed this step in a structured model selection and evaluation approach, based on a specific statistical framework [29].

PTS investigated only a 5-hidden-state ARHMM, as suggested by the topology clustering. It is likely that for larger datasets, representing more heterogenous populations (e.g. multi morbidity cohorts), the number of hidden states could be much higher and as a result more complex trajectories can be discovered.

Pseudo-time analysis results validation and comparison with a reference time line depends from the clinical problem, the data availability and the left censoring strategies. In the current study we use the follow-up time from the first at the hospital to assess whether the trajectories correctly model patient progression. While the progression into stages drawn by pseudo-time is independent by the first observation, a better time reference to depict the stages of a disease progression might be the time form diagnosis.

Our approach has a clear application in precision medicine, especially for chronic diseases like T2DM. Temporal phenotypes can be exploited to compare responses to therapies and to find novel biomarkers that are able to discern responses during the disease’s progression. Some examples are already available, where TDA has been used for classification purposes [30], and it can be exploited to illustrate deviations in the disease space drawn from probabilities of moving forward into nodes with higher density of complications.

Another important advancement in the analysis of temporal trajectories would compare treatments for individual patients and their possible use for comparing disease deviations or adverse outcomes [[31], [32], [33], [34]], which could be naturally integrated into clinical decision support systems. Our approach could facilitate the integration of temporal phenotypes into these systems thanks to its methodological rigor and the possibility of studying continuous transitions among states, but also to the possibility of visually delivering and explaining its results in a clear and understandable way to researchers and clinicians.

## Declaration of Competing Interest

None.
